# Zymomonas mobilis ZM4 Utilizes an NADP^+^-Dependent Acetaldehyde Dehydrogenase To Produce Acetate

**DOI:** 10.1128/jb.00563-21

**Published:** 2022-03-08

**Authors:** Magdalena M. Felczak, Michaela A. TerAvest

**Affiliations:** a Department of Biochemistry and Molecular Biology, Michigan State University, East Lansing, Michigan, USA; University of Florida

**Keywords:** *Zymomonas*, bioenergy, enzymes, fermentation, metabolism

## Abstract

Zymomonas mobilis is a promising bacterial host for biofuel production, but further improvement has been hindered because some aspects of its metabolism remain poorly understood. For example, one of the main by-products generated by Z. mobilis is acetate, but the pathway for acetate production is unknown. Acetaldehyde oxidation has been proposed as the major source of acetate, and an acetaldehyde dehydrogenase was previously isolated from Z. mobilis via activity guided fractionation, but the corresponding gene has never been identified. We determined that the locus ZMO1754 (also known as ZMO_RS07890) encodes an NADP^+^-dependent acetaldehyde dehydrogenase that is responsible for acetate production by Z. mobilis. Deletion of this gene from the chromosome resulted in a growth defect in oxic conditions, suggesting that acetaldehyde detoxification is an important role of acetaldehyde dehydrogenase. The deletion strain also exhibited a near complete abolition of acetate production, both in typical laboratory conditions and during lignocellulosic hydrolysate fermentation. Our results show that ZMO1754 encodes the major acetate-forming acetaldehyde dehydrogenase in Z. mobilis, and we therefore rename the gene *aldB* based on functional similarity to the Escherichia coli acetaldehyde dehydrogenase.

**IMPORTANCE** Biofuel production from nonfood crops is an important strategy for reducing carbon emissions from the transportation industry, but it has not yet become commercially viable. An important avenue to improve biofuel production is to enhance the characteristics of fermentation organisms by decreasing by-product formation via genetic engineering. Here, we identified and deleted a metabolic pathway and associated gene that lead to acetate formation in Zymomonas mobilis. Acetate is one of the major by-products generated during ethanol production by Z. mobilis, so this information may be used in the future to develop better strains for commercial biofuel production.

## INTRODUCTION

Zymomonas mobilis is an alphaproteobacterium that produces ethanol from glucose with a very high specificity and may be useful for biofuel production (1). Under optimal conditions, Z. mobilis converts glucose to ethanol at up to 97% of the theoretical yield, converting only a small fraction of the carbon into by-products or biomass (2, 3). However, under less-favorable conditions, Z. mobilis generates by-products, including acetaldehyde and acetate, especially when exposed to oxygen (4–6). Stressors present in lignocellulosic hydrolysates may also lead to by-product formation. We previously observed that Z. mobilis produces 5 to 10 mM acetate when grown in anaerobic lignocellulosic hydrolysate (7). Acetate production during lignocellulose fermentation is problematic because it reduces the efficiency of biofuel production and further increases the acetate concentration. Acetate is formed by many biomass hydrolysis methods, leading to high acetate concentrations in lignocellulosic hydrolysates, which are often inhibitory to the fermentation organism (8, 9). Because of the already inhibitory concentrations and low value of acetate, generation of this molecule during hydrolysate fermentations is highly undesirable.

**FIG 1:**
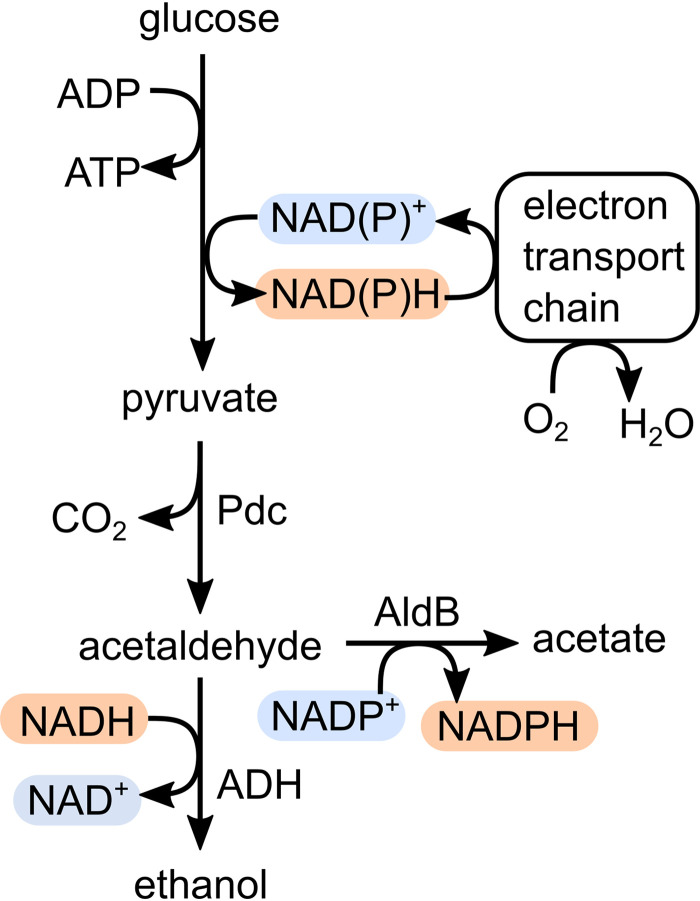
Central metabolism of Z. mobilis, including the acetaldehyde dehydrogenase encoded by *aldB*. Oxidized redox cofactors are highlighted in blue and reduced redox cofactors are highlighted in red to indicate the connections formed between oxygen and ethanol, acetaldehyde, and acetate formation. Pdc, pyruvate decarboxylase; ADH, alcohol dehydrogenases I and II.

Although acetate production by Z. mobilis has been well-documented, the metabolic pathway that leads to significant acetate secretion is unknown (10). One possible source of acetate secreted by Z. mobilis is oxidation of acetaldehyde, which is an intermediate in the ethanol production pathway. Acetaldehyde is typically undetectable in anoxic cultures of Z. mobilis due to fast reduction by ethanol-forming alcohol dehydrogenases, ADHI and ADHII. However, acetaldehyde accumulates in oxic cultures because NAD(P)H that is oxidized by the respiratory chain is not available to reduce acetaldehyde to ethanol (Fig. 1). Acetaldehyde is inhibitory to Z. mobilis even at low concentrations (0.05%, wt/wt) and is considered the main contributor to poor growth of Z. mobilis in oxic conditions (11, 12). Acetate is much better tolerated (up to 0.8%, wt/wt); therefore, oxidation of acetaldehyde to acetate provides a possible route for detoxification with additional NAD(P)H being recycled by the respiratory chain (13).

An NADP^+^-dependent acetaldehyde dehydrogenase was discovered in Z. mobilis strain Z6 in 1989 via activity guided fractionation, suggesting that acetaldehyde oxidation could be a major source of acetate (14). However, there is no acetaldehyde dehydrogenase annotated in the Z. mobilis Z6 genome (15). Further, we have not found an annotated acetaldehyde dehydrogenase in any other Z. mobilis genome (7). As a result, most genome scale metabolic models for Z. mobilis only include acetate as a by-product of less central reactions, such as amino acid biosynthesis (10, 16, 17). Only one model includes acetaldehyde dehydrogenase, although no gene evidence was provided for the reaction (18). Metabolic models of Z. mobilis tend to underestimate acetate production compared with experimental results (10), suggesting that a major source of acetate is missing. To address this gap in our knowledge of Z. mobilis metabolism, we identified a locus in the Z. mobilis ZM4 genome encoding an acetaldehyde dehydrogenase and determined that it is the main source of acetate production under laboratory growth conditions and during lignocellulosic hydrolysate fermentation. Here, we refer to the locus ZMO1754 as *aldB* due to the functional similarity to the E. coli gene of the same name. The ZM4 strain was used because it is the most promising strain for hydrolysate fermentation (7), and recently developed genome modification tools facilitated generation of a markerless deletion strain in this background (19).

## RESULTS

To determine which gene in the Z. mobilis ZM4 genome encodes an ortholog of the previously purified acetaldehyde dehydrogenase from Z6, we searched for a locus with the following characteristics: (i) the gene should encode a protein with an approximate size of 55 kDa, and (ii) the gene should be induced under aerobic conditions (14). Based on a recent multi-omic analysis of oxygen exposure in Z. mobilis ZM4 by Martien et al. (20), we hypothesized that locus ZMO1754 (also known as ZMO_RS07890) encodes the previously discovered acetaldehyde dehydrogenase. ZMO1754 encodes a protein with an expected size of 49.6 kDa, and both its transcript and protein abundance increased upon oxygen exposure (20). ZMO1754 is annotated as an NAD^+^-dependent succinate-semialdehyde dehydrogenase, suggesting a role as a redox enzyme. Further, an ortholog of ZMO1754 is present in the Z6 genome, indicating that it could encode the previously identified acetaldehyde dehydrogenase. The results below show that the protein encoded by ZMO1754 shares strong functional similarity to the E. coli gene *aldB*, which is also an NADP^+^-dependent acetaldehyde dehydrogenase (21). Due to the functional similarity, we propose to rename the locus ZMO1754 to *aldB* in Z. mobilis and refer to the gene as *aldB* throughout the manuscript.

**FIG 2:**
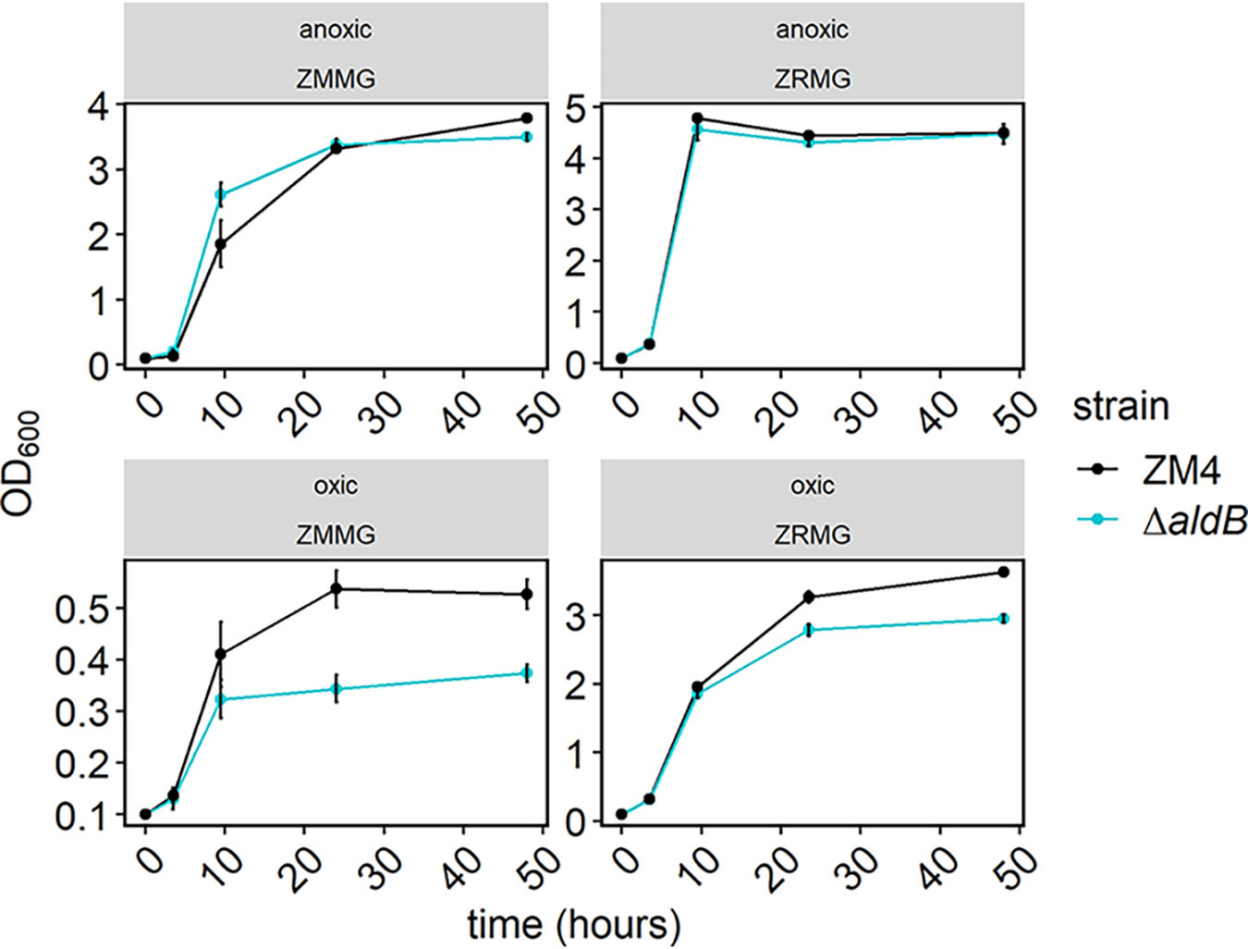
Growth of Z. mobilis ZM4 WT (black) and the Δ*aldB* strain (blue). Overnight cultures were inoculated from single colonies and grown overnight in ZRMG medium (1% yeast extract, 15 mM phosphate buffer, 2% glucose) or minimal ZMMG medium (mineral salts, 0.1% phosphate buffer, 2% glucose, calcium pantothenate) in static cultures in oxic or anoxic conditions. For subsequent anoxic growth, the cultures were diluted to an OD_600_ of 0.1 in 5 mL of fresh rich or minimal medium in Hungate tubes in an anaerobic chamber, and tubes were closed with stoppers and secured with crimps. For oxic growth, cultures were started similarly but Hungate tubes were covered with aluminum foil. All cultures were grown outside anaerobic chamber in 30°C with shaking at 250 rpm for the time indicated. Samples were taken periodically to monitor growth. Points represent the average of 3 biological replicates, and error bars represent standard error. Note that *y* axis scales differ across panels.

**FIG 3:**
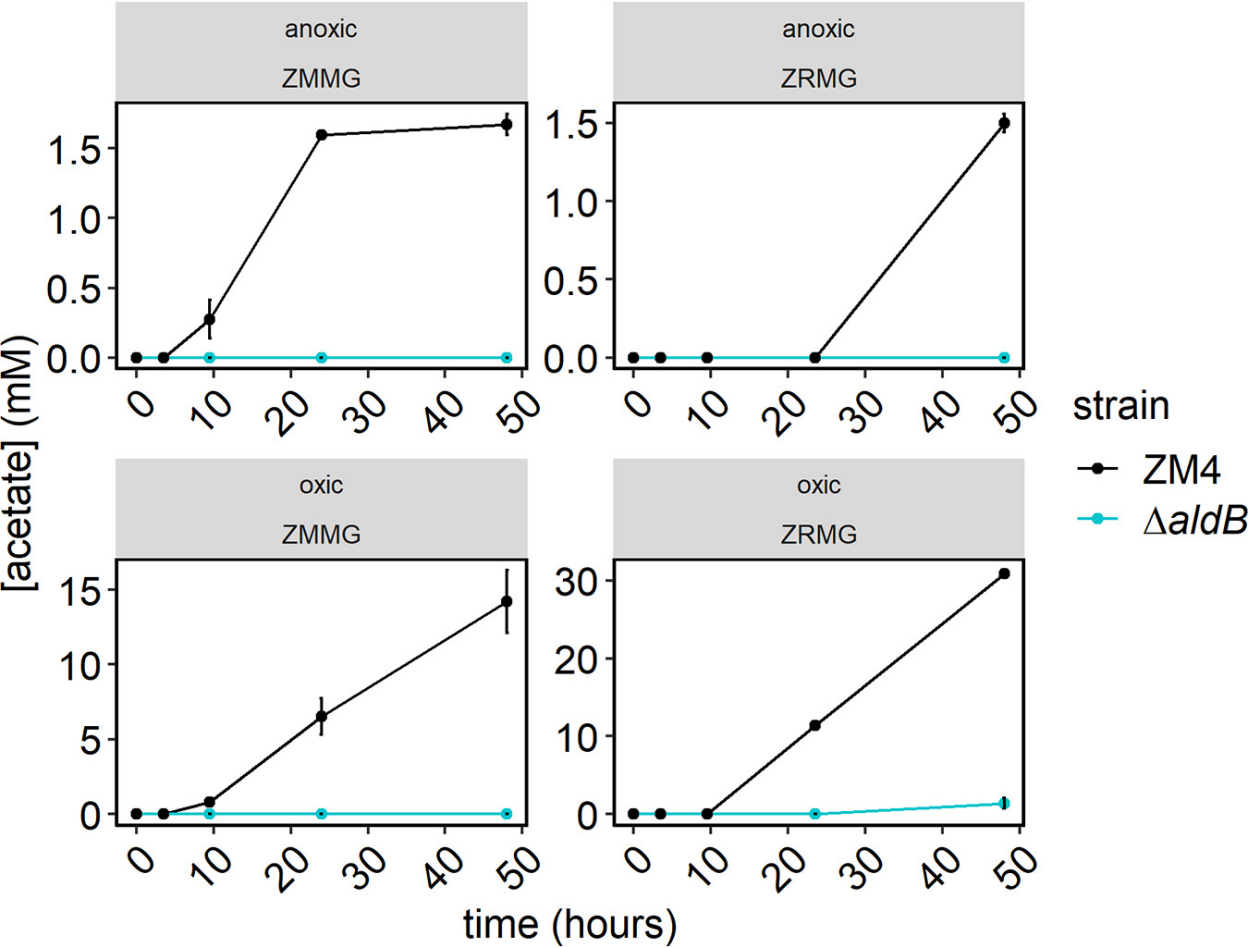
Acetate production by Z. mobilis ZM4 WT (black) and the Δ*aldB* strain (blue). Samples from cultures used in Fig. 2 were centrifuged, and clear supernatants were analyzed by HPLC as described in Materials and Methods. Acetate concentrations were calculated from standard curves. Points represent the average of 3 biological replicates, and error bars represent standard error. Note that *y* axis scales differ across panels.

To determine whether *aldB* encodes an acetaldehyde dehydrogenase, we first deleted it from the Z. mobilis ZM4 genome and observed the effect on growth in oxic and anoxic conditions in rich and minimal media (Fig. 2). We observed that the deletion had little impact on growth in anoxic conditions but reduced the final culture density by 19% and 30% in oxic rich and minimal media, respectively. We also measured acetate production by both strains under each condition. The wild-type (WT) strain produced acetate in all culture conditions, while acetate production by the Δ*aldB* strain was undetectable except in oxic-rich medium (Fig. 3). In oxic-rich medium, the Δ*aldB* strain produced a small amount of acetate (1.37 mM), but this was a 96% decrease compared with the 30.87 mM produced by WT under the same conditions.

**FIG 4:**
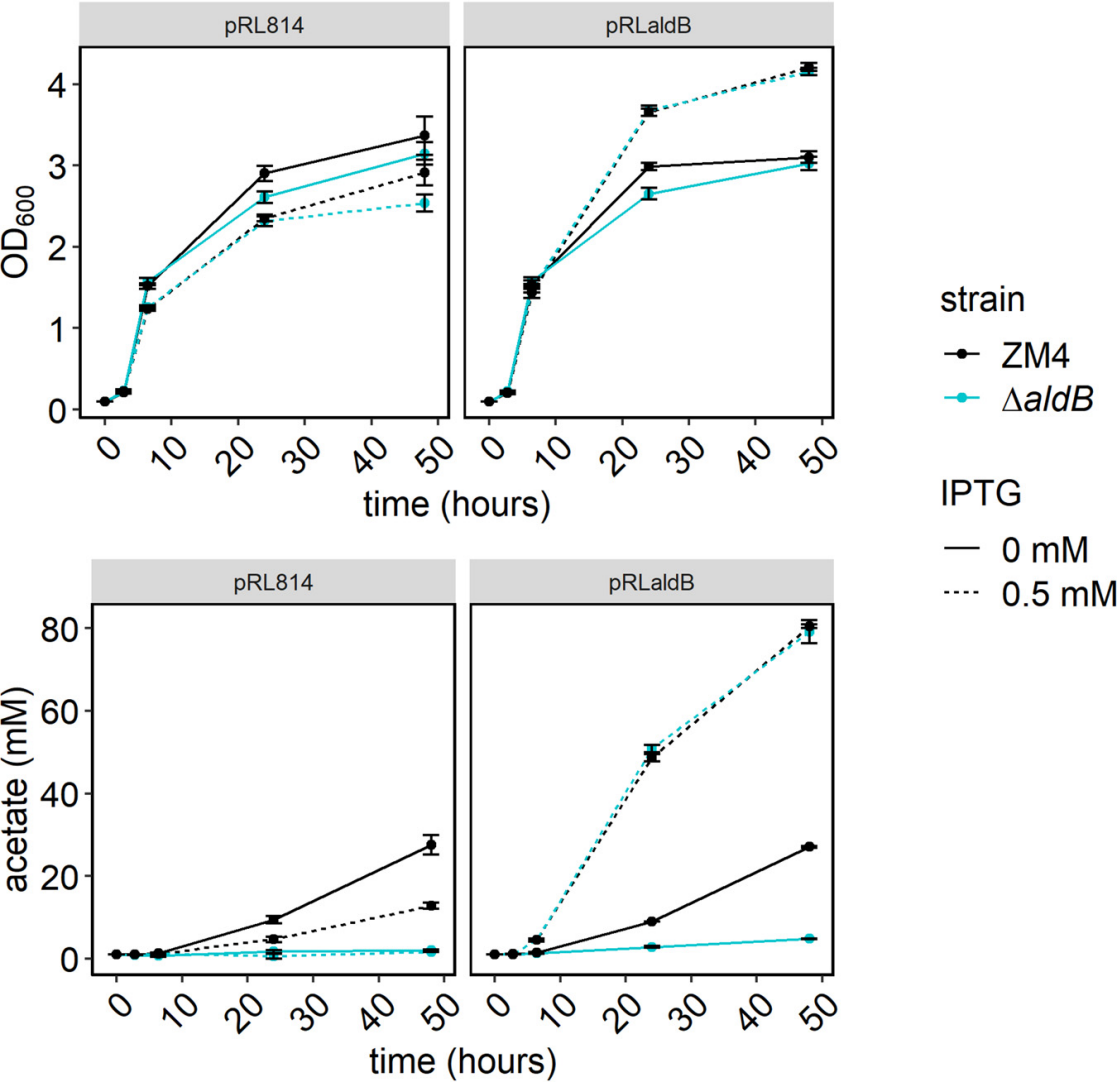
Growth and acetate production by Z. mobilis ZM4 WT (black) and the Δ*aldB* strain (blue) bearing plasmid pRL*aldB* with (dashed) and without (solid) IPTG induction. Strains bearing pRL*aldB* or pRL814 were grown as in Fig. 2, but media were supplemented with 100 μg/mL of spectinomycin and IPTG was added to 0.5 mM, at time of dilution, when indicated. HPLC analysis was performed as in Fig. 3. Points represent the average of three biological replicates, and error bars represent standard error.

We complemented the Δ*aldB* strain by expressing *aldB* from an isopropyl-β-d-thiogalactopyranoside (IPTG)-inducible plasmid (pRL*aldB*) and measuring growth and acetate production in oxic-rich medium (Fig. 4). We used pRL814, which expresses green fluorescent protein (GFP) under an IPTG-inducible promoter as a control for the effect of the plasmid and heterologous protein synthesis. We observed that expressing *aldB* dramatically improved growth for both strains while expressing *gfp* slightly reduced growth. *aldB* expression increased acetate production by nearly 3-fold for ZM4 and over 16-fold for the Δ*aldB* strain, while *gfp* expression had no effect on acetate production. Overall, we found that expression of *aldB* in *trans* complemented the growth and acetate production phenotypes of the Δ*aldB* strain. Further, expression in *trans* increased growth and acetate production in WT under the conditions tested. We also performed complementation with His-tagged AldB and observed the same results, indicating that the tagged version of the protein is active (see Fig. S3 in the supplemental material).

We confirmed that the Δ*aldB* phenotype was caused by loss of acetaldehyde dehydrogenase activity by using an activity assay to compare soluble protein fractions of the WT and Δ*aldB* strain. We tested activity of the soluble protein fraction to convert acetaldehyde to acetate using NADP^+^ or NAD^+^ as the cofactor. We observed that deletion of *aldB* completely abolished the NADP^+^-dependent activity. In WT, the NAD^+^-dependent acetaldehyde dehydrogenase activity was <25% as high as the NADP^+^-dependent activity (Table 1; see also Fig. S4 in the supplemental material). The NAD^+^-dependent activity was reduced slightly by deletion of *aldB*.

**TABLE 1:** Acetaldehyde dehydrogenase activity in soluble protein fractions from the ZM4 and Δ*aldB* strains^*a*^

Strain	Cofactor	Activity^*b*^	Relative activity (%)^*b*^
ZM4 WT	NADP^+^	0.18 ± 0.04	1.00
Δ*aldB* mutant	NADP^+^	N.D.^*c*^	N.D.
ZM4 WT	NAD^+^	0.04 ± 0.01	0.24
Δ*aldB* mutant	NAD^+^	0.03 ± 0.00	0.17

a Soluble protein fraction (FI) was obtained as described in Materials and Methods. Each enzymatic reaction of 1 mL contained 0.1 M Tris HCl pH 8.0, 0.1 M KCl, 10 mM β-mercaptoethanol, 2 mM acetaldehyde, and 0.67 mM NAD^+^ or NADP^+^ (protocol for yeast acetaldehyde dehydrogenase from Sigma-Aldrich). Reaction was started by adding 33 μl of FI and measured for 30 min at 25°C in 24-well microtiter plate using plate reader. Absorbance at 340 nm in control without FI was subtracted from the reactions.

b Activity was calculated as increase in NAD(P)H concentration/min/mL per milligram of total protein in FI. Millimolar extinction coefficient for NAD(P)H in 1 mL of solution in 24-well plate was determined experimentally as 4.35. Values represent the average and standard deviation from three independent experiments with three technical repeats.

c N.D., not detected.

We next overexpressed a His-tagged version of AldB in E. coli and confirmed acetaldehyde dehydrogenase activity of the tagged protein in the soluble protein fraction from cells induced with IPTG (see Fig. S5 in the supplemental material). We purified the protein using Ni-affinity chromatography as described in Materials and Methods (see Fig. S6 in the supplemental material). We pooled fractions based on their specific activity and used fractions 9 to 11 (specific activity, 23.3 U/mg protein) to measure substrate and cofactor specificity using an acetaldehyde dehydrogenase assay as described in Materials and Methods. We observed that the activity was specific for acetaldehyde with moderate activity toward other short-chain fatty aldehydes, propionaldehyde (3C), butyraldehyde (4C), and valeraldehyde (5C) (Table 2). The activity toward formaldehyde and glyceraldehyde was low. Because the previous annotation of *aldB* was “succinate semialdehyde dehydrogenase,” we also tested succinate semialdehyde as a substrate. The activity toward succinate semialdehyde was only 10% of the activity with acetaldehyde. AldB was also specific for NADP^+^ as a cofactor, showing only 18% relative activity with NAD^+^ as the cofactor.

**TABLE 2:** Substrate specificity of ZM4 acetaldehyde dehydrogenase^*a*^

Substrate	Cofactor	Relative activity^*b*^
Acetaldehyde	NADP^+^	100
Acetaldehyde	NAD^+^	18 ± 2
Succinate semialdehyde	NADP^+^	10 ± 1.2
Propionaldehyde	NADP^+^	64 ± 15
Butyraldehyde	NADP^+^	83 ± 10
Valeraldehyde	NADP^+^	47 ± 10
Formaldehyde	NADP^+^	4 ± 3
Glyceraldehyde	NADP^+^	8 ± 3

a Enzymatic reactions were performed in standard assay conditions (22 mM aldehyde, 2 mM NADP^+^ or NAD^+^, 10 mM DTT, 50 mM Tris HCl pH 8.0, and 50 mU of enzyme at 30°C as described in Materials and Methods for 30 min). Aldehydes were added as 0.23 M solutions in dimethyl sulfoxide (DMSO).

b Activity was normalized by setting activity with acetaldehyde at 100%. Data are averages of 3 to 5 independent experiments with three technical replicates for each substrate.

**FIG 5:**
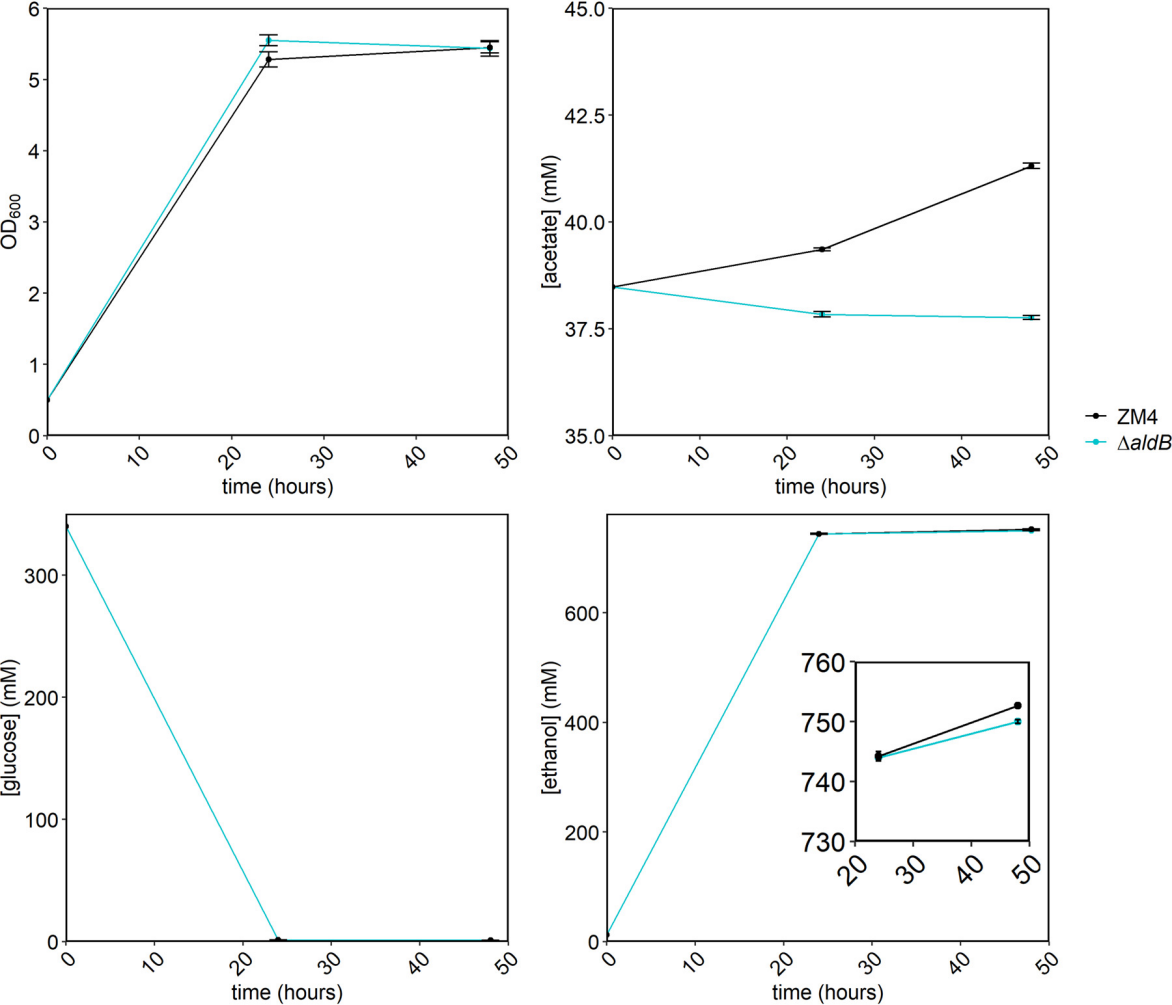
Growth and HPLC analysis of Z. mobilis ZM4 WT (black) and the Δ*aldB* strain (blue) in AFEX hydrolysate. Overnight cultures were grown in ZRMG medium at 30°C in static cultures in anoxic conditions. 7% AFEX hydrolysate was inoculated to an OD of 0.5 as described in Materials and Methods, and cultures were incubated in Hungate tubes outside the chamber at 30°C with shaking. Samples were taken after 24 and 48 h for OD_600_ and HPLC analysis. OD_600_ was measured in cultures diluted 10 times in water. Samples for HPLC analysis were prepared as in Fig. 3. Points represent the average of 3 biological replicates, and error bars represent standard error.

Because we previously observed some acetate secretion by ZM4 grown in lignocellulosic hydrolysate (7), we also measured growth and acetate concentration in fermentations using ZM4 or the Δ*aldB* strain (Fig. 5). Growth and substrate utilization by the two strains was not significantly different; both strains reached a final optical density at 600 nm (OD_600_) of 5.4 after 48 h and consumed all available glucose. However, the acetate and ethanol concentrations were significantly different between the two strains. The initial acetate concentration in the hydrolysate was 38.48 mM, and after 48 h of fermentation by ZM4, the concentration rose to 41.31 ± 0.06 mM. In contrast, with the Δ*aldB* strain, the acetate concentration decreased slightly to 37.76 ± 0.04 mM. Ethanol production by the Δ*aldB* strain was slightly decreased at 750.01 ± 0.28 mM versus 752.7 ± 0.21 mM for ZM4.

## DISCUSSION

We determined that the locus ZMO1754 (also known as ZMO_RS07890) in the Z. mobilis ZM4 genome encodes an acetaldehyde dehydrogenase that is the main source of acetate production. Here, we refer to the gene encoded at that locus as *aldB* based on functional similarity to E. coli *aldB*. Although acetate production by Z. mobilis is well-documented and an acetaldehyde dehydrogenase was previously purified from strain Z6, the genes necessary for acetate production were previously unknown. Yang et al. previously speculated that ZMO1754 could encode an acetaldehyde dehydrogenase but did not test the hypothesis because they found no evidence of upregulation in response to oxygen (5). However, a more recent transcriptomic and proteomic characterization of oxygen response in Z. mobilis did observe that the transcript and protein encoded by ZMO1754 were upregulated in response to oxygen (20). The newer multi-omic characterization led us to hypothesize that ZMO1754 encodes an acetaldehyde dehydrogenase. Our deletion studies show that ZMO1754 encodes the enzyme responsible for acetate production in Z. mobilis ZM4.

We also characterized AldB from Z. mobilis ZM4 to determine whether it is an ortholog of the NADP^+^-dependent acetaldehyde dehydrogenase purified from Z. mobilis Z6 by Barthel et al. (14). We found that both are aldehyde dehydrogenases with significant specificity toward acetaldehyde, are upregulated in response to oxygen, have approximately the same molecular weight, and use NADP^+^ rather than NAD^+^ as a cofactor. Although we observed a minor NAD^+^-dependent acetaldehyde dehydrogenase activity in the protein fraction extracted from the Δ*aldB* strain, both acetate concentrations in culture and activity assays confirm that *aldB* encodes the major acetaldehyde dehydrogenase.

The presence of a growth defect in the Δ*aldB* strain only under oxic conditions suggests that acetate production is beneficial only when oxygen is present. This finding aligns with previous research indicating that acetaldehyde accumulation by Z. mobilis occurs only when oxygen is present (Fig. 1) (11, 22). Although there was some acetate production under anoxic conditions, the concentration was >10-fold lower than in oxic conditions, suggesting that there is little acetaldehyde available as a substrate when oxygen is absent. Acetaldehyde concentrations in Z. mobilis are kept low under anoxic conditions by the ethanol-forming alcohol dehydrogenases, ADHI and ADHII. Although ADH and AldB compete for acetaldehyde as a substrate, flux to ethanol versus acetate is likely controlled by the availability of NAD(P)H and NAD(P)^+^. Under anoxic conditions, even if acetaldehyde is oxidized, the resulting NADPH can only be recycled by ethanol production. Only when oxygen is present and the respiratory chain becomes active can NADPH be recycled, allowing significant flux toward acetate production. In context with previous work, our findings suggest that the main role of AldB is to detoxify acetaldehyde that is generated under oxic conditions.

While deletion of *aldB* resulted in a dramatic reduction in NADP^+^-dependent acetaldehyde dehydrogenase activity in the soluble protein fraction, the NAD^+^-dependent acetaldehyde dehydrogenase activity was changed only slightly. This observation suggests that another enzyme in Z. mobilis is capable of oxidizing acetaldehyde to acetate using NAD^+^ as the cofactor. We hypothesize that this enzyme is responsible for the residual acetate production observed in the Δ*aldB* strain in rich medium under oxic conditions (Fig. 3). At this time, it is impossible to say which enzyme is responsible for this activity, although based on the low amount of acetate produced, we speculate that it may be off-target activity of a dehydrogenase that is more active with another aldehyde.

From a biotechnological perspective, identifying the locus encoding acetaldehyde dehydrogenase is useful because a deletion strain could be used to eliminate acetate production in biofuel fermentations. Conversely, we observed decreased ethanol production by the Δ*aldB* strain, suggesting that acetate production may be involved in a stress-response pathway in Z. mobilis ZM4. We speculate that reducing equivalents may be diverted from central metabolism to stress response pathways that modify inhibitory small molecules found in lignocellulosic hydrolysates. The diversion of reducing equivalents would necessitate acetaldehyde detoxification as observed in oxic conditions. Further, Z. mobilis has been proposed as a host for acetaldehyde production, and deletion of *aldB* is likely to dramatically increase acetaldehyde yield (12, 23). (Note that we do not report acetaldehyde concentrations here because our culture method resulted in significant evaporative loss of acetaldehyde.) Further work is necessary to determine whether deletion or upregulation of *aldB* from Z. mobilis would be beneficial for biotechnological processes overall.

We expect our findings to be generalizable across *Zymomonas* because the acetaldehyde dehydrogenase encoded by *aldB* in strain ZM4 appears to be conserved across the entire *Zymomonas* genus. We recently conducted a genome comparison analysis for all sequenced *Zymomonas* genomes and used our previous ortholog analysis to determine that an *aldB* ortholog is present in all analyzed genomes. The AldB protein shares 99% amino acid identity across Z. mobilis subspecies *mobilis* and 93% and 84% between Z. mobilis *francensis* and Z. mobilis *mobilis* and Z. mobilis *pomaceae* and Z. mobilis *mobilis*, respectively (see Fig. S7 in the supplemental material) (7). A BLAST search of the AldB sequence against the ZM4 genome shows <30% identity for any protein other than AldB, indicating that this protein did not likely arise from a gene duplication in the *Zymomonas* genome. In contrast, a BLAST search against all bacteria shows proteins with >50% identity in other alphaproteobacteria, including *Bradyrhizobium* spp. and *Paraburkholderia* spp., suggesting that *aldB* was present in common ancestors of *Zymomonas* and other bacterial genera. Further research is needed to determine whether AldB also functions as an NADP^+^-dependent acetaldehyde dehydrogenase in other bacterial species.

## MATERIALS AND METHODS

### Media and chemicals.

Escherichia coli strains were grown in Luria-Bertani medium (Miller; Acumedia). Zymomonas mobilis ZM4 was grown in ZRMG (1% yeast extract, 2% glucose, and 15 mM KH_2_PO_4_) or ZMMG [1.0 g KH_2_PO_4,_ 1.0 g K_2_HPO_4,_ 0.5 g NaCl, 1.0 g (NH_4_)_2_SO_4_, 0.2 g MgSO_4_ · 7 H_2_O, 0.025 g Na_2_MoO_4_ · 2 H_2_O, 0.025 g FeSO_4_ · 7 H_2_O, 0.010 g CaCl_2_ · 2 H_2_O, 0.001 g calcium pantothenate, 20.0 g d-glucose per 1 L]. Solid medium contained 1.5% agar (Difco). Chloramphenicol was added to 100 μg/mL or 35 μg/mL and spectinomycin to 100 μg/mL or 50 μg/mL for Z. mobilis and E. coli, respectively. Ampicillin was used at 100 μg/mL for E. coli. 2,6-Diaminopimelic acid (DAP; Aldrich) was added to a final concentration of 0.3 mM when needed. Seven percent glucan ammonia-fiber expansion (AFEX) switchgrass hydrolysate (ASGH) from biomass grown in 2016 was produced by the Great Lakes Bioenergy Research Center core facility (24). B-PER reagent was from Thermo Scientific, and Profinity immobilized metal affinity chromatography (IMAC) Ni-charged resin was from Bio-Rad; all IMAC buffers contained 20 mM Tris HCl pH 8, 0.3 M NaCl, 10% glycerol, and imidazole at 5 mM, 20 mM, or 500 mM for IMAC A, B, and C, respectively. Complete EDTA-free protease inhibitor tablets were from Sigma-Aldrich/Roche. Bradford protein assay reagents 1 and 2 and Precision Plus Protein all blue prestained protein standard were from Bio-Rad. The anti His-tag mouse antibody was from GenScript (catalog no. A00186). Horseradish peroxidase-conjugated rabbit anti-mouse antibody was from Sigma-Aldrich. Tris-buffered saline with Tween 20 (TBST) buffer is 20 mM Tris-HCl pH 7.5, 150 mM NaCl, 0.01% Tween 20. Clarity Western ECL was from Bio-Rad. NADP^+^ sodium salt was from Sigma-Aldrich. NAD^+^ was from Amresco. Acetaldehyde, propionaldehyde, butyraldehyde, valeraldehyde, formaldehyde, and glyceraldehyde were from Sigma-Aldrich/Millipore. Succinate semialdehyde (15% solution) was from Alfa Chemistry. Restriction enzymes, T4 ligase, Q5DNA polymerase and HiFi DNA assembly master mix were from New England BioLabs. Egg lysozyme was from Sigma-Aldrich, and IPTG was from GoldBio. Oligonucleotides were synthesized by Integrated DNA Technologies (IDT).

### Bacterial strains and plasmids.

Zymomonas mobilis ATCC 31821 (ZM4) was obtained from Robert Landick (University of Wisconsin, Madison). E. coli strains Mach 1 and BL21(DE3) pLysS were from Invitrogen. E. coli WM6026 (*lacIq rrnB3* Δ*lacZ*4787 *hsdR*514Δ*araBAD*567 Δ*rhaBAD568* rph-1 attλ::pAE12(ΔoriR6K-cat::Frt5), ΔendA::Frt uidA(ΔMluI)::pir attHK::pJK1006(ΔoriR6K-cat::Frt5; trfA::Frt) ΔdapA::Frt [25]) was obtained from Patricia Kiley (University of Wisconsin, Madison). The ZM4 Δ*aldB* strain was constructed using the method described in Lal et al. (19). Briefly, 500-bp fragments directly upstream and downstream of *aldB* were PCR amplified with Q5 DNA polymerase using the following pairs of primers: “ZMO1754 up” for upstream and “ZMO1754 dn” for downstream fragments (Table 3). The fragments were cloned into the SpeI restriction site of a nonreplicating plasmid pPK15534 (19), digested with SpeI, using NEB Builder HiFi DNA assembly master mix to get pPKΔZMO1754 (see Fig. S1 in the supplemental material). The insertion was confirmed by PCR. pPKΔZMO1754 was introduced into ZM4 by conjugation from WM6026/pPKΔZMO1754 and subsequent selection for chloramphenicol resistance and DAP independence, as described in Lal et al. (19). Integration of pPKΔZMO1754 into the chromosome by homologous recombination in chloramphenicol-resistant cells was confirmed by colony PCR. One colony with inserted plasmid was grown for about 10 generations in liquid ZRMG medium without chloramphenicol, and 10,000 CFU were plated from an appropriate dilution onto ZRMG plates to get around 100 colonies per plate. Plates were incubated for at least 48 h in aerobic conditions at 30°C, and colonies were screened for loss of green fluorescence under a blue light illuminator. Nonfluorescent colonies were checked for loss of *aldB* by PCR using primers *aldB* up F and *aldB* dn R (Table 3), followed by Sanger sequencing.

**TABLE 3:** Oligonucleotides^*a*^

Name	Sequence	Description
ZMO1754 up F	TCATGTTTGACAGCTTATCATTCTGGTTCACAGCGTCC	F primer to amplify 500 bp upstream of *aldB*
ZMO1754 up R	AGGCTCTTTTAAATTTCTTAAAATCCGTTTTAAGCG	R primer to amplify 500 bp upstream of *aldB*
ZMO1754 dn F	TAAGAAATTTAAAAGAGCCTACCTTTCTTATTACTTCAGCTTAGGAC	F primer to amplify 500 bp downstream of *aldB*
ZMO1754 dn R	CAAGGCAAGACCGAGCGCTAGGTGTGGACGGGCGGTGA	R primer to amplify 500 bp downstream of *aldB*
*aldB* up F	GCCGATCTGGATTTCAGACCG	61 bp upstream of *aldB*
*aldB* dn R	GGAGGACTGTCCTAAGCTGAAG	45 downstream of *aldB*
MF_9	GGAGATATACATATGGCATATGAATCTGTCAATCCCG	F primer; amplifies *aldB* with overlaps to pRL814
MF_10	ATATCAAGCTTAGGCAGCAGAAGCGTCTATCAA	R primer; amplifies *aldB* with overlaps to pRL814
MF_11	GCTGCCTAAGCTTGATATCGAATTCCTGCAGC	F primer; amplifies pRL814 with overlaps to *aldB*
MF_12	TTCATATGCCATATGTATATCTCCTTCTTAAAGTTAAACTAATTCTAGATGTG	R primer; amplifies pRL814 with overlaps to *aldB*
MF_17	AGGTCGTCATATGGCATATGAATCTGTCAATCCCG	F primer; amplifies *aldB* with overlaps to pET16B
MF_18	CCGGATCCTTAGGCAGCAGAAGCGTCTATCAATT	R primer; amplifies *aldB* with overlaps to pET16B
MF_19	ATATGCCATATGACGACCTTCGATATGGCC	F primer; amplifies pET16b with overlaps for *aldB*
MF_20	CTGCCTAAGGATCCGGCTGCTAACAAAG	R primer; amplifies pET16b with overlaps for *aldB*
MF_23	ATATCAAGCTTAGGCAGCAGAAGCGTCTATCA	F primer; amplifies His-*aldB* on pET16b*aldB*
MF_24	AGATATACATATGGGCCATCATCATCATCATCATCATCATCATCAC	R primer; amplifies His-*aldB* on pET16b*aldB*
MF_22	GCTGCCTAAGCTTGATATCGAATTCCTGCAGCC	F primer; amplifies pRL814 for GA of His-AldB
MF_21	TGATGGCCCATATGTATATCTCCTTCTTAAAGTTAAACTAATTCTAGATGTGT	R primer; amplifies pRL814 for GA of His-AldB

a F, forward; R, reverse; GA, Gibson assembly.

Oligonucleotides and plasmids are listed in Table 3 and Table 4, respectively. pET16b*aldB* was constructed by amplification of *aldB* from ZM4 genomic DNA using primers MF_17 and MF_18 and Gibson assembly into the pET16b amplified from primers MF_19 and MF_20. This assembly places *aldB* between BamHI and NdeI of pET16b under the control of the T7 promoter and downstream of a 10-histidine tag (see Fig. S2 in the supplemental material). After transformation into Mach 1, plasmid was isolated from ampicillin-resistant colonies, and the sequence was confirmed by Sanger sequencing. Next, pET16b*aldB* was transformed to BL21(DE3) pLysS, and IPTG-induced synthesis of His-tagged AldB was confirmed by Western blotting as described in the His-AldB purification section. pRL*aldB* was constructed by amplification of *aldB* from ZM4 using primers MF_9 and MF_10 and assembled with pRL814 amplified with primers MF_11 and MF_12 using NEB HiFi DNA assembly master mix. This assembly replaces the GFP gene in pRL814 with *aldB*. pRLHis-*aldB* was constructed by amplification of His-*aldB* from pET16b*aldB* using primers MF_23 and MF_24 and Gibson assembly with the pRL814 backbone amplified with primers MF_21 and MF_22. After transformation into Mach 1, isolated plasmids were sequenced by the Sanger method.

**TABLE 4:** Plasmids

Plasmid	Parent	Description	Relevant genotype	Reference or source
pPK15534		Suicide vector	GFP, Cm^r^, SpeI site	19
pPKΔZMO1754	pPK15534	ZMO1754 (*aldB*) knockout plasmid	GFP, Cm^r^, 1,000 bp insert up/down *aldB*	This work
pRL814		Broad host range plasmid	T7 *lac* GFP, Sp^r^	26
pRL*aldB*	pRL814	Complementing plasmid	T7 *lac aldB*, Sp^r^	This work
pRLHis-*aldB*	pRL814	Complementing plasmid	T7 *lac* His-*aldB*, Sp^r^	This work
pET16b		E. coli expression vector	His-tag, Amp^r^	Novagen
pET16b*aldB*	pET16b	*aldB* expression in E. coli	His-*aldB*, Amp^r^	This work

### Bacterial growth.

The ZM4 or Δ*aldB* strain were grown in ZRMG or ZMMG at 30°C, with shaking at 250 rpm. For anaerobic conditions, bacteria were grown in Hungate tubes: 5 mL of media stored in an anaerobic chamber (Coy Lab Products) were inoculated from single colonies in the chamber, and the tubes were closed with butyl-rubber stoppers and secured with aluminum crimps before removing from the chamber. For aerobic growth, Hungate tubes were loosely covered with aluminum foil. Samples were removed periodically for OD_600_ measurements and high-pressure liquid chromatography (HPLC) analysis. Spectinomycin was added to cultures bearing pRL814, pRL*aldB*, or pRLHis-*aldB*, and IPTG was added as indicated.

### AFEX hydrolysate fermentation.

pH of hydrolysate was adjusted to 5.8 with 10 M NaOH. The hydrolysate was resterilized by filtration and stored in an anaerobic chamber. Ten milliliters of ZRMG was inoculated from fresh ZRMG plates of Z. mobilis ZM4 WT or the Δ*aldB* strain, and cultures were grown anaerobically, overnight to an OD_600_ of approximately 3 in the anaerobic chamber. Cultures were concentrated to an OD_600_ of 10 by centrifuging cells at 8,000 rpm for 10 min (Sorval ST8; Thermo Scientific) at room temperature and resuspending in an appropriate volume of the hydrolysate. Five milliliters of hydrolysate in Hungate tubes was inoculated to an OD_600_ of 0.5 under anaerobic chamber, and tubes were capped and sealed. Tubes were incubated outside the chamber at 30°C with shaking for 50 h. Samples were taken after 24 and 48 h for OD_600_ and HPLC analysis. For OD_600_ measurements, samples were diluted 10 times in H_2_O. Samples for HPLC analysis were stored at −20°C.

### HPLC analysis.

Samples were prepared by centrifugation at 14,000 rpm for 10 min in microfuge tubes at 4°C to remove cells, and supernatants were transferred into 2-mL glass vials. Analysis was performed on Shimadzu 20A HPLC using an Aminex HPX-87H (Bio-Rad) column at 50°C. Separation of compounds was with 5 mM sulfuric acid at a flow rate of 0.6 mL/minute. Compounds were identified by refractive index and concentrations determined from standard curves for external standards run in the same batch.

### Soluble protein fraction from Z. mobilis.

Fifty milliliters ZRMG in 250-mL flasks were inoculated from fresh colonies of ZM4 or the Δ*aldB* strain and grown aerobically with shaking at 250 rpm at 30°C to an OD_600_ of 1.0. Cells were harvested by centrifugation at 4°C at 8,000 rpm for 10 min (Sorval ST 8R 75005709 rotor). Pellets were washed twice with 2 mL ice-cold 0.1 M Tris HCl pH 8.0 and resuspended in 0.25 mL of the same buffer. Lysis components were added to the following final concentrations: 20 mM EDTA, 1 mg/mL lysozyme, 4 mM dithiothreitol (DTT), and protease inhibitor as recommended by manufacturer. Lysis was performed at 37°C for 20 min. Samples were spun in microfuge tubes at 17,000 rpm (Sorval ST 8R 75005715 rotor) for 30 min at 4°C. Clear supernatant (soluble protein fraction [FI]) was gently transferred to new microfuge tubes and flash frozen in liquid nitrogen. FI was stored at −80°C.

### Purification of His-AldB by Ni-affinity chromatography.

One hundred milliliters of LB containing ampicillin was inoculated from a single colony of E. coli BL21(DE3) pLysS bearing pET16b*aldB* and grown at 30°C with shaking at 250 rpm overnight. An overnight culture was diluted to an OD_600_ of 0.1 in 2 L of LB medium with ampicillin in a 6-L flask and grown with shaking at 30°C to an OD_600_ of 0.6. At this point, IPTG was added to a final concentration of 0.1 mM, and growth was continued for 2 h to an OD_600_ of 2.5. Cells were harvested by centrifugation at 6,000 rpm for 10 min (GC-3 Sorval) at 4°C. Pellets were resuspended gently in 20 mL of ice-cold B-PER reagent containing two tablets of complete protease inhibitor and flash frozen in liquid nitrogen. On the next day, cells were thawed on ice, and solid NaCl was added to a final concentration of 0.3 M and 100 mM imidazole to 5 mM. At this point, cells were completely lysed. The soluble fraction was separated from membranes and cell debris by ultracentrifugation (Beckman; Ti 45) at 22,000 rpm for 40 min. Clear supernatant, containing soluble protein fraction (FI), was analyzed by SDS-PAGE for the presence of His-AldB, and the total protein concentration was determined using a Bradford assay. Activity of His-AldB was measured by an acetaldehyde dehydrogenase assay as described below. Eleven milliliters of FI containing 14 mg/mL of total protein was mixed with 2.5 mL of Ni-charged IMAC resin equilibrated with IMAC buffer A and incubated on a rocker for 1 h in 4°C. The entire slurry was loaded onto a 10-mL Thermo-Fisher disposable column under gravity flow at 4°C. The column was washed with 10 column volumes (CV) of IMAC A and 10 CV of IMAC B under gravity flow. His-AldB was eluted with 5 CV of IMAC C; 0.25-mL fractions were collected. Protein concentration in fractions was determined by the Bradford method, and the purity of His-AldB was estimated by SDS-PAGE followed by Coomassie staining and Western blotting with anti-His antibody. Dithiothreitol (DTT) was added to a final concentration of 10 mM to the peak fractions before freezing in liquid nitrogen. Fractions were stored at −80°C.

### SDS-PAGE and Western blotting.

The indicated amounts of Ni-chromatography fraction of His-*aldB* or soluble protein fraction was added to gel loading buffer (0.4 M Tris base, 30% glycerol, 1% SDS, 0.5% bromophenol blue), containing 10 mM DTT to get a final volume of 10 μl and boiled for 5 min. To get whole-cell lysate, a pellet from 1 mL of cells at an OD_600_ of 1.0 was resuspended in 0.1 mL of loading buffer and boiled for 10 min. Samples were loaded on 4 to 20% gradient Mini-Protean TGX stain-free gels (Bio-Rad) and run in Tris/glycine/SDS running buffer (Bio-Rad). Gels were stained with Coomassie brilliant blue, or proteins were transferred into nitrocellulose membranes in Trans-Blot Turbo transfer buffer (Bio-Rad) for 7 min in a Trans-Blot Turbo transfer system (Bio-Rad). Membranes were blocked with 3% bovine serum albumin (BSA) in TBST and incubated with mouse anti-His monoclonal antibody overnight in a cold room. Membranes were washed in TBST buffer for 2 h and incubated with horseradish peroxidase-conjugated rabbit anti-mouse antibody for 2 h. The membranes were washed, and chemiluminescent detection was performed with Clarity Western ECL (Bio-Rad) for 5 min.

### Standard acetaldehyde dehydrogenase assay.

Reactions were performed *in vitro* in 1 mL of reaction buffer in 24-well plate using a Synergy H1 BioTek plate reader. The standard reaction buffer contained 50 mM Tris pH 8.0, 10 mM DTT, 22 mM acetaldehyde, 2 mM NADP^+^ or NAD^+^, and 50 mU of purified His-AldB or indicated volumes of soluble protein fraction from Z. mobilis or E. coli expressing *aldB* from plasmid. The reaction was performed at 30°C for 30 min. Acetaldehyde dehydrogenase activity was measured as reduction of NAD^+^/NADP^+^ to NADH/NADPH by monitoring absorbance at 340 nm. One unit of enzyme is the amount that catalyzes formation of 1 μmol acetate per minute.
