# Supplementary material for: Zymomonas mobilis ZM4 Utilizes an NADP+-Dependent Acetaldehyde Dehydrogenase To Produce Acetate
Source: J Bacteriol. 2022 Mar 8;204(4):e00563-21. doi: 10.1128/jb.00563-21 (PMC9017298; doi:10.1128/jb.00563-21)
Supplement: SUPPLEMENTAL FILE 1 — Fig. S1 to S7. Download jb.00563-21-s0001.pdf, PDF file, 0.6 MB [file jb.00563-21-s0001.pdf]

Supplementary Figures

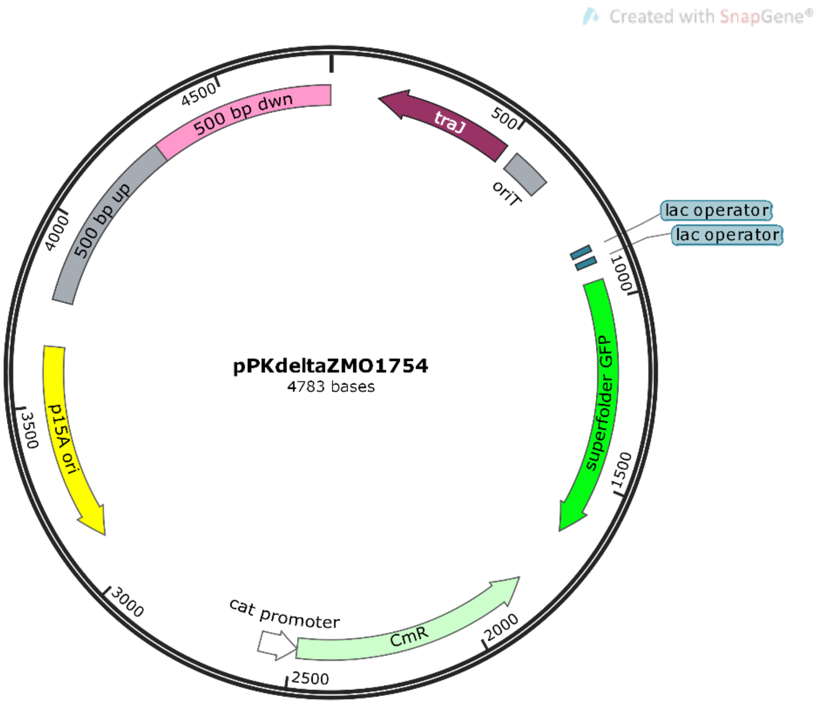

Figure S1. pPKΔZMO1754 map. 500 bp fragments directly upstream and downstream of ZMO1754 were amplified from ZM4 chromosome and Gibson assembly into pPK15534 digested with SpeI as described in Materials and Methods.

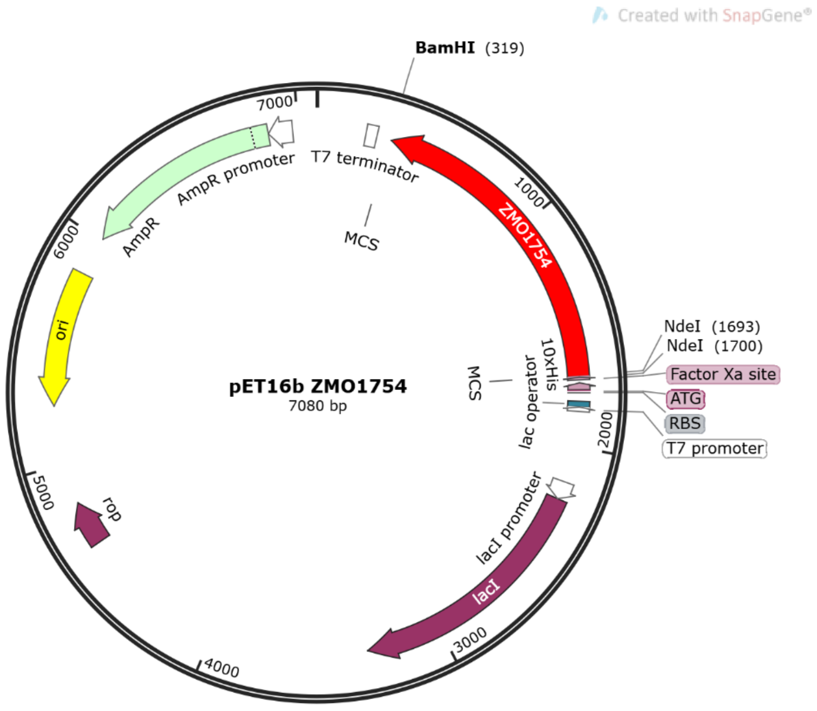

Figure S2. pET16b*aldB* map. The *aldB* (ZMO1754) gene was amplified from ZM4 and cloned to pET16b between NdeI and BamHI sites using Gibson Assembly as described in Materials and Methods. AldB protein was fused with 10 histidine residues at N-terminus and placed under T7 promoter of pET16b. Primers are listed in Table 2.

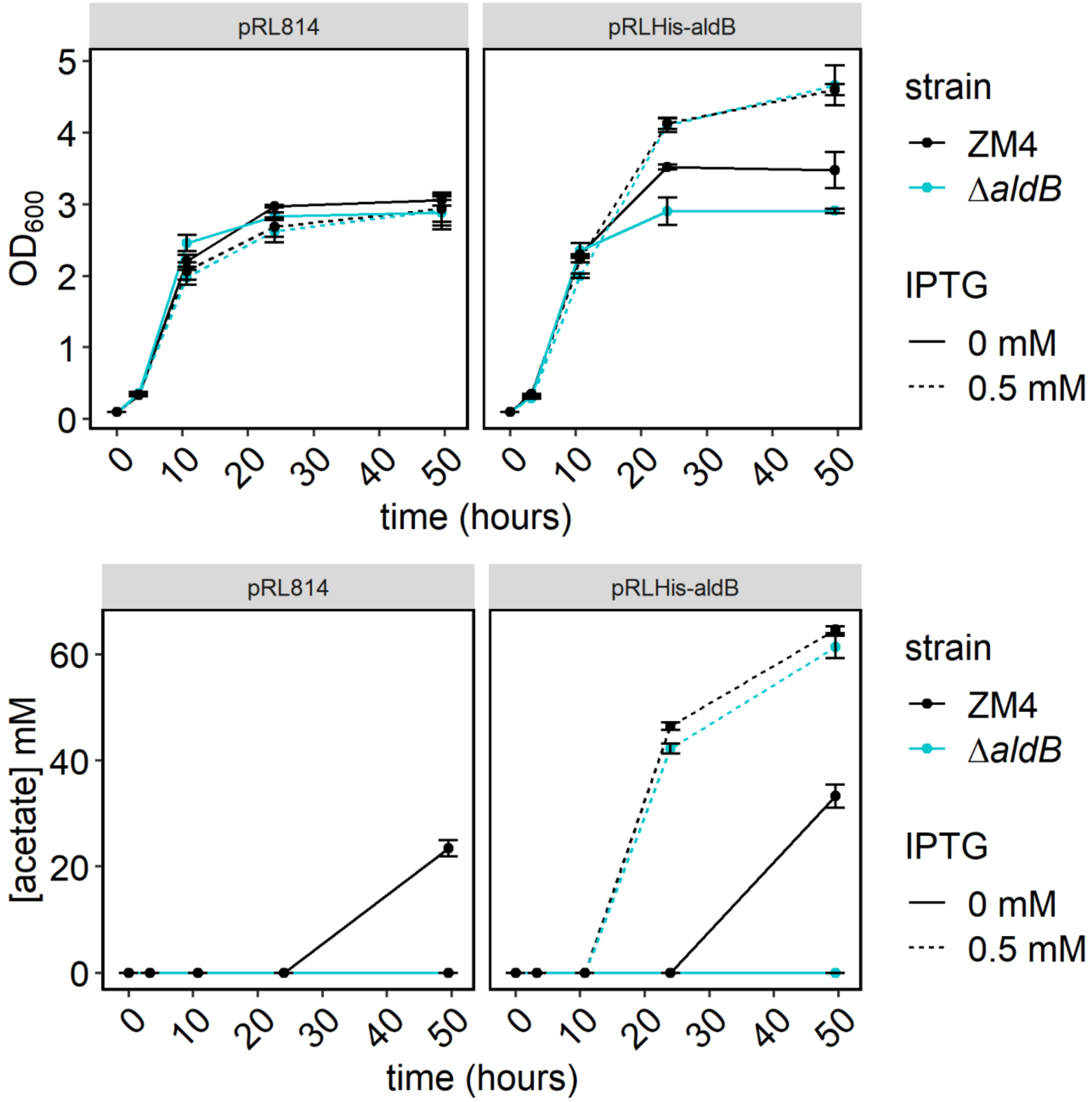

Figure S3. Growth and acetate production by *Z. mobilis* ZM4 WT (black) and Δ*aldB* (blue) bearing plasmid pRLHis-*aldB* with (dashed) and without (solid) IPTG induction. Strains bearing pRLHis-*aldB* or pRL814, were grown as in Figure 2 but media were supplemented with 100 g/ml of spectinomycin and IPTG was added to 0.5 mM, at time of dilution, when indicated. HPLC analysis was performed as in Figure 3 Points represent the average of three biological replicates and error bars represent standard error.

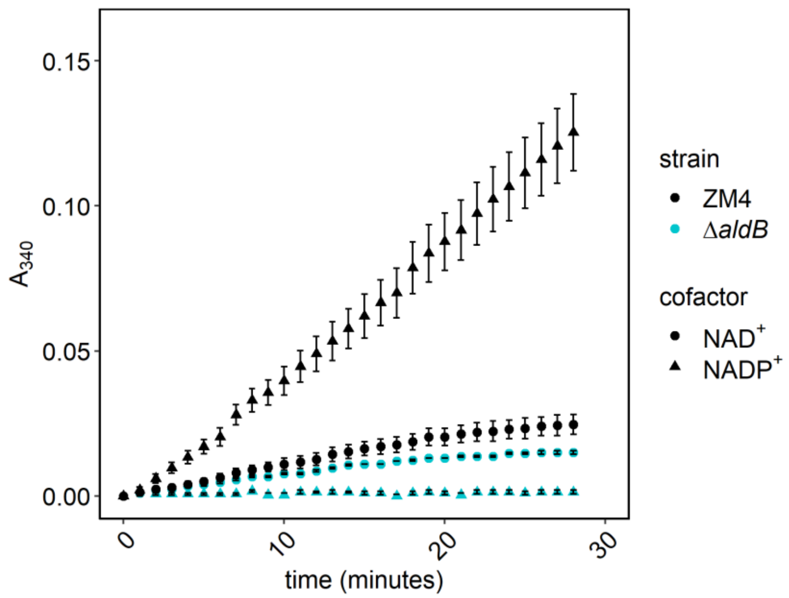

Figure S4. Acetaldehyde dehydrogenase activity in soluble protein fractions from ZM4 WT and Δ*aldB*. Soluble protein fraction (FI) was obtained as described in “Materials and Methods”. Average, total protein concentration in FI was 5.7 +/- 0.5 mg/ml and 5.2 +/- 0.7 mg/ml for ZM4 and Δ*aldB*, respectively. Each enzymatic reaction contained: 0.1 M Tris HCl pH 8.0, 0.1 M KCl, 10 mM β-mercaptoethanol, 2 mM acetaldehyde and 0.67 mM NAD^+ or $NADP^+$ (protocol for yeast acetaldehyde dehydrogenase from Sigma-Aldrich). Reaction was started by adding 33 □l of FI and measured for 30 minutes at 25°C in 24-well microtiter plate. Absorbance at 340 nm in control without FI was subtracted from the reactions. Points represent the average of three independent experiments with three technical repeats and error bars represent standard error.

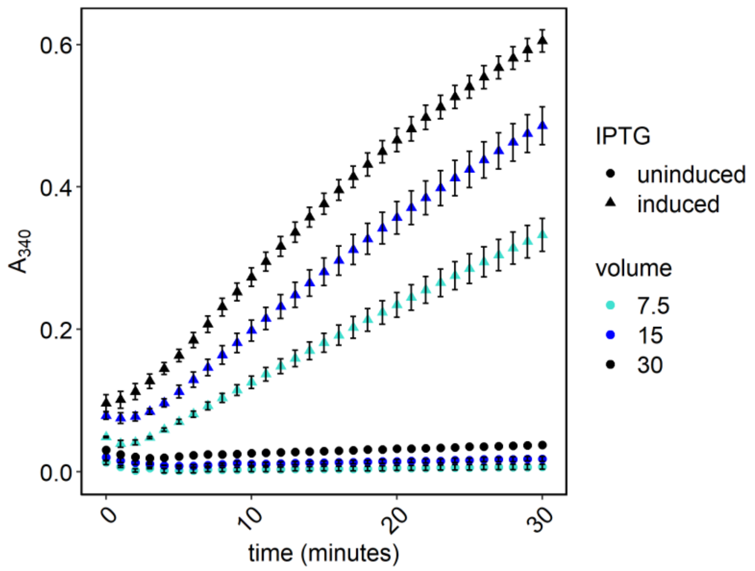

Figure S5. Acetaldehyde dehydrogenase activity in soluble protein fraction from *E. coli* BL21 carrying a plasmid expressing *aldB*. 50-mL cultures were grown in LB supplemented with ampicillin at 30ºC to OD_{600} 0.4. At this point, IPTG was added to a final concentration of 0.1 mM, where indicated, and growth continued for 1.5 hours. FI was obtained as described in “Materials and Methods” for *Z. mobilis*. Average total protein concentration in three independent FI was 11±1.2 mg/ml and 8.4±1.1 mg/ml for uninduced and induced cultures, respectively. An enzymatic assay was performed as described in Figure S4, with NADP as a cofactor. The reaction was started by adding variable volumes of FI as indicated. Points represent the average of three independent FI and error bars represent standard error.

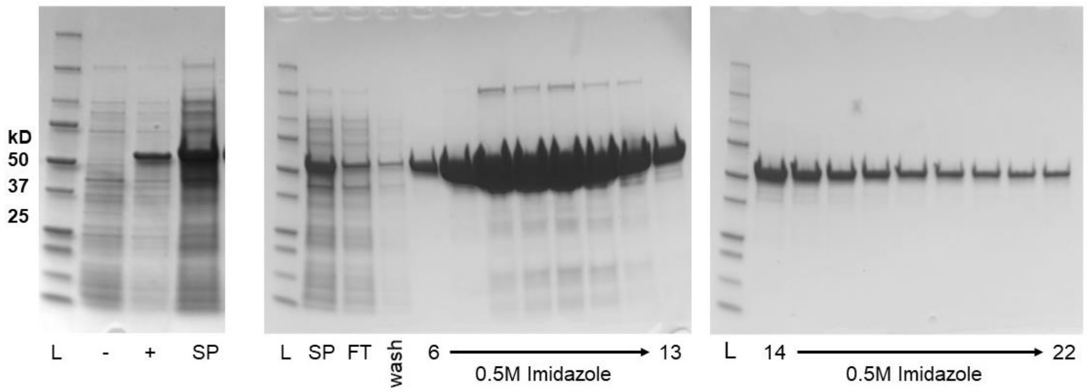

Figure S6. Ni-affinity purification of His-AldB. Protein was overexpressed in *E. coli* BL21(DE3) from pET16b*aldB* induced with IPTG as described in Materials and Methods. Soluble protein fraction was loaded on Ni-affinity column and the protein was eluted with IMAC C buffer containing 0.5 M imidazole. Protein on different stages of purification is visualized on Comassie stained SDS-PAGE as described in Materials and Methods. L, All Blue Protein Ladder; -/+ indicates induction with IPTG; SP, soluble protein fraction; FT, flow through; wash, IMAC B (20 mM imidazole buffer); 6-22, IMAC C fractions.

```
CLUSTAL multiple sequence alignment by MUSCLE (3.8)
pomaceae        MAYESVNPATGETVKKYPDLSDAQVKEAIDRTFDVFQKDWGKRSIEDRSKILHKAAEIFR
francensis      MAYESVNPATGETVKKYPSFSDAQVKEAVDRAATVFKNDWSQRTIAERSKVLHKAADIFR
Z6              MAYESVNPATGEIVKKYPDFSDKQVKESVDRAATVFKNDWSQRTIAERSKVLHKAAEIFR
B23394          MAYESVNPATGEIVKKYPDFSDKQVKESVDRAATVFKNDWSQRTIAERSKVLHKAAEIFR
B4492           MAYESVNPATGEIVKKYPDFSDKQVKESVDRAATVFKNDWSQRTIAERSKVLHKAAEIFR
ZM4             MAYESVNPATGETVKKYPDFSDKQVKDSVDRAATVFKNDWSQRTIAERSKVLHKAAEIFR
CP1             MAYESVNPATGETVKKYPDFSDKQVKDSVDRAATVFKNDWSQRTIAERSKVLHKAAEIFR
B1960           MAYESVNPATGETVKKYPDFSDKQVKDSVDRAATVFKNDWSQRTIAERSKVLHKAAEIFR
CP3             MAYESVNPATGETVKKYPDFSDKQVKDSVDRAATVFKNDWSQRTIAERSKVLHKAAEIFR
CP4             MAYESVNPATGETVKKYPDFSDKQVKDSVDRAATVFKNDWSQRTIAERSKVLHKAAEIFR
B12526          MAYESVNPATGETVKKYPDFSDKQVKDSVDRAATVFKNDWSQRTIAERSKVLHKAAEIFR
B10988          MAYESVNPATGETVKKYPDFSDKQVKDSVDRAATVFKNDWSQRTIAERSKVLHKAAEIFR
CU1             MAYESVNPATGETVKKYPDFSDKQVKDSVDRAATVFKNDWSQRTIAERSKVLHKAAEIFR
CU1rif2         MAYESVNPATGETVKKYPDFSDKQVKDSVDRAATVFKNDWSQRTIAERSKVLHKAAEIFR
uvs51           MAYESVNPATGETVKKYPDFSDKQVKDSVDRAATVFKNDWSQRTIAERSKVLHKAAEIFR
PROIMI          MAYESVNPATGETVKKYPDFSDKQVKDSVDRAATVFKNDWSQRTIAERSKVLHKAAEIFR
NCIMB11163      MAYESVNPATGETVKKYPDFSDKQVKDSVDRAATVFKNDWSQRTIAERSKVLHKAAEIFR
                ************ *****.:** ***:::**:  **::**.:*:* :***:*****:***

pomaceae        SDIDKYARLMTLEMGKRIEEARGEVKLSADILDYYAKNGSTFLAPQKVEEKPGAVVKAFP
francensis      SDIDKYAKLMTIEMGKRLEEARGEVKLSADILDYYAKNGAAFLAPQKVEEKPGAVIKAFP
Z6              SDVDKYAKLLTIDMGKKIAEARGEVKLSADILDYYAKNGEKFLAPQKVEEKPGAVVKAFP
B23394          SDVDKYAKLLTIDMGKKIAEARGEVKLSADILDYYAKNGEKFLAPQKVEEKPGAVVKAFP
B4492           SDVDKYAKLLTIDMGKKIAEARGEVKLSADILDYYAKNGEKFLAPQKVEEKPGAVVKAFP
ZM4             SDVDKYAKLLTIDMGKKIAEARGEVNLSADILDYYAKNGEKFLAPQKVEEKPGAVVKAFP
CP1             SDVDKYAKLLTIDMGKKIAEARGEVKLSADILDYYAKNGEKFLAPQKVEEKPGAVVKAFP
B1960           SDVDKYAKLLTIDMGKKIAEARGEVKLSADILDYYAKNGEKFLAPQKVEEKPGAVVKAFP
CP3             SDVDKYAKLLTIDMGKKIAEARGEVKLSADILDYYAKNGEKFLAPQKVEEKPGAVVKAFP
CP4             SDVDKYAKLLTIDMGKKIAEARGEVKLSADILDYYAKNGEKFLAPQKVEEKPGAVVKAFP
B12526          SDVDKYAKLLTIDMGKKIAEARGEVKLSADILDYYAKNGEKFLAPQKVEEKPGAVVKAFP
B10988          SDVDKYAKLLTIDMGKKIAEARGEVKLSADILDYYAKNGEKFLAPQKVEEKPGAVVKAFP
CU1             SDVDKYAKLLTIDMGKKIAEARGEVKLSADILDYYAKNGEKFLAPQKVEEKPGAVVKAFP
CU1rif2         SDVDKYAKLLTIDMGKKIAEARGEVKLSADILDYYAKNGEKFLAPQKVEEKPGAVVKAFP
uvs51           SDVDKYAKLLTIDMGKKIAEARGEVKLSADILDYYAKNGEKFLAPQKVEEKPGAVVKAFP
PROIMI          SDVDKYAKLLTIDMGKKIAEARGEVKLSADILDYYAKNGEKFLAPQKVEEKPGAVVKAFP
NCIMB11163      SDVDKYAKLLTIDMGKKIAEARGEVKLSADILDYYAKNGEKFLAPQKVEEKPGAVVKAFP
                **:****.*:*::***.: ******:*************  **************:****

pomaceae        LGLILAIEPWNFPYYQLARIAGPYLVAGNALLVKHSSSVPQCAHAFEEVLEQAGVPKGAY
francensis      LGLLLAIEPWNFPYYQLARIAGPYLVAGNALLVKHSSSVPQSAHAFEAVLEEAGAPKGIY
Z6              LGLLLAIEPWNFPYYQLARIAGPYLIAGNALLVKHSSSVPQSAHAFEAVLEEAGAPKGIY
B23394          LGLLLAIEPWNFPYYQLARIAGPYLIAGNALLVKHSSSVPQSAHAFEAVLEEAGAPKGIY
B4492           LGLLLAIEPWNFPYYQLARIAGPYLIAGNALLVKHSSSVPQSAHAFEAVLEEAGAPKGIY
ZM4             LGLLLAIEPWNFPYYQLARIAGPYLIAGNALLVKHSSSVPQSAHAFEAVLEEAGAPKGIY
CP1             LGLLLAIEPWNFPYYQLARIAGPYLIAGNALLVKHSSSVPQSAHAFEAVLEEAGAPKGIY
B1960           LGLLLAIEPWNFPYYQLARIAGPYLIAGNALLVKHSSSVPQSAHAFEAVLEEAGAPKGIY
CP3             LGLLLAIEPWNFPYYQLARIAGPYLIAGNALLVKHSSSVPQSAHAFEAVLEEAGAPKGIY
CP4             LGLLLAIEPWNFPYYQLARIAGPYLIAGNALLVKHSSSVPQSAHAFEAVLEEAGAPKGIY
B12526          LGLLLAIEPWNFPYYQLARIAGPYLIAGNALLVKHSSSVPQSAHAFEAVLEEAGAPKGIY
B10988          LGLLLAIEPWNFPYYQLARIAGPYLIAGNALLVKHSSSVPQSAHAFEAVLEEAGAPKGIY
CU1             LGLLLAIEPWNFPYYQLARIAGPYLIAGNALLVKHSSSVPQSAHAFEAVLEEAGAPKGIY
CU1rif2         LGLLLAIEPWNFPYYQLARIAGPYLIAGNALLVKHSSSVPQSAHAFEAVLEEAGAPKGIY
uvs51           LGLLLAIEPWNFPYYQLARIAGPYLIAGNALLVKHSSSVPQSAHAFEAVLEEAGAPKGIY
PROIMI          LGLLLAIEPWNFPYYQLARIAGPYLIAGNALLVKHSSSVPQSAHAFEAVLEEAGAPKGIY
NCIMB11163      LGLLLAIEPWNFPYYQLARIAGPYLIAGNALLVKHSSSVPQSAHAFEAVLEEAGAPKGIY
                ***:*********************:***************.***** ***:**.*** *
```

```
pomaceae        TNLDASPAQVSSLIEDSRVRGVTVTGSASVGAELAAKAGKMWKKSVMELGGSDAFIVLEG
francensis      TNLDASPDQISQIIEDPRVRGVTVTGSASVGAELAAKAGKMWKKSVMELGGSDAFIVLDG
Z6              TNLDASPDQISQIIEDPRVRGVTVTGSASVGAELAAKAGKMWKKSVMELGGSDAFIVLDG
B23394          TNLDASPDQISQIIEDPRVRGVTVTGSASVGAELAAKAGKMWKKSVMELGGSDAFIVLDG
B4492           TNLDASPDQISQIIEDPRVRGVTVTGSASVGAELAAKAGKMWKKSVMELGGSDAFIVLDG
ZM4             TNLDASPDQVSQIIEDPRVRGVTVTGSASVGAELAAKAGKMWKKSVMELGGSDAFIVLDG
CP1             TNLDASPDQVSQIIEDPRVRGVTVTGSASVGAELAAKAGKMWKKSVMELGGSDAFIVLDG
B1960           TNLDASPDQVSQIIEDPRVRGVTVTGSASVGAELAAKAGKMWKKSVMELGGSDAFIVLDG
CP3             TNLDASPDQVSQIIEDPRVRGVTVTGSASVGAELAAKAGKMWKKSVMELGGSDAFIVLDG
CP4             TNLDASPDQVSQIIEDPRVRGVTVTGSASVGAELAAKAGKMWKKSVMELGGSDAFIVLDG
B12526          TNLDASPDQVSQIIEDPRVRGVTVTGSASVGAELAAKAGKMWKKSVMELGGSDAFIVLDG
B10988          TNLDASPDQVSQIIEDPRVRGVTVTGSASVGAELAAKAGKMWKKSVMELGGSDAFIVLDG
CU1             TNLDASPDQVSQIIEDPRVRGVTVTGSASVGAELAAKAGKMWKKSVMELGGSDAFIVLDG
CU1rif2         TNLDASPDQVSQIIEDPRVRGVTVTGSASVGAELAAKAGKMWKKSVMELGGSDAFIVLDG
uvs51           TNLDASPDQVSQIIEDPRVRGVTVTGSASVGAELAAKAGKMWKKSVMELGGSDAFIVLDG
PROIMI          TNLDASPDQVSQIIEDPRVRGVTVTGSASVGAELAAKAGKMWKKSVMELGGSDAFIVLDG
NCIMB11163      TNLDASPDQVSQIIEDPRVRGVTVTGSASVGAELAAKAGKMWKKSVMELGGSDAFIVLDG
                ******* *:*.:***.*****************************************:*

pomaceae        VDIDDKLIEQAVYGRLFNAGQVCCAAKRFIIVGKKQAEIFTEKLKARFESLKIGDPFDEK
francensis      VDIDDKLIEKAAYGRLFNAGQVCCAAKRFIIVGEKRAELFTEKLKQRFESLKIGDPMDES
Z6              VDIDDKLIDKAAYGRLFNAGQVCCAAKRFIIVGQKRAELFTEKLKQRFEALKIGDPMDES
B23394          VDIDDKLIDKAAYGRLFNAGQVCCAAKRFIIVGQKRAELFTEKLKQRFEALKIGDPMDES
B4492           VDIDDKLIDKAAYGRLFNAGQVCCAAKRFIIVGQKRAELFTEKLKQRFEALKIGDPMDES
ZM4             VDIDDKLIDKAAYGRLFNAGQVCCAAKRFIIVGQKRAELFTEKLKQRFEALKIGDPMDES
CP1             VDIDDKLIDKAAYGRLFNAGQVCCAAKRFIIVGQKRAELFTEKLKQRFEALKIGDPMDES
B1960           VDIDDKLIDKAAYGRLFNAGQVCCAAKRFIIVGQKRAELFTEKLKQRFEALKIGDPMDES
CP3             VDIDDKLIDKAAYGRLFNAGQVCCAAKRFIIVGQKRAELFTEKLKQRFEALKIGDPMDES
CP4             VDIDDKLIDKAAYGRLFNAGQVCCAAKRFIIVGQKRAELFTEKLKQRFEALKIGDPMDES
B12526          VDIDDKLIDKAAYGRLFNAGQVCCAAKRFIIVGQKRAELFTEKLKQRFEALKIGDPMDES
B10988          VDIDDKLIDKAAYGRLFNAGQVCCAAKRFIIVGQKRAELFTEKLKQRFEALKIGDPMDES
CU1             VDIDDKLIDKAAYGRLFNAGQVCCAAKRFIIVGQKRAELFTEKLKQRFEALKIGDPMDES
CU1rif2         VDIDDKLIDKAAYGRLFNAGQVCCAAKRFIIVGQKRAELFTEKLKQRFEALKIGDPMDES
uvs51           VDIDDKLIDKAAYGRLFNAGQVCCAAKRFIIVGQKRAELFTEKLKQRFEALKIGDPMDES
PROIMI          VDIDDKLIDKAAYGRLFNAGQVCCAAKRFIIVGQKRAELFTEKLKQRFEALKIGDPMDES
NCIMB11163      VDIDDKLIDKAAYGRLFNAGQVCCAAKRFIIVGQKRAELFTEKLKQRFEALKIGDPMDES
                ********::*.*********************:*.**:****** ***:******:**.

pomaceae        TTLAPLSSIAARDQVVAQVEKAVSNGATLVCGGKALKRPGAFMEAGILTDISRDNPAYFE
francensis      TQLGPLSSTGARDQIVEQVEKAVKNGAKLVCGGKAIKGKGAFMEAGILTNIKRDNPAYFE
Z6              TDLGPLSSVGARDQVVKQVEKAVQNGAKLVCGGKAIEGKGAFMKAGILTNIKRENPAYFE
B23394          TDLGPLSSVGARDQVVKQVEKAVQNGAKLVCGGKAIEGKGAFMKAGILTNIKRENPAYFE
B4492           TDLGPLSSVGARDQVVKQVEKAVQNGAKLVCGGKAIEGKGAFMKAGILTNIKRENPAYFE
ZM4             TDLGPLSSVGARDQVVKQVEKAVQNGAKLVCGGKAIEGKGAFMKAGILTDIKRENPAYFE
CP1             TDLGPLSSVGARDQVVKQVEKAVQNGAKLVCGGKAIEGKGAFMKAGILTDIKRENPAYFE
B1960           TDLGPLSSVGARDQVVKQVEKAVQNGAKLVCGGKAIEGKGAFMKAGILTDIKRENPAYFE
CP3             TDLGPLSSVGARDQVVKQVEKAVQNGAKLVCGGKAIEGKGAFMKAGILTDIKRENPAYFE
CP4             TDLGPLSSVGARDQVVKQVEKAVQNGAKLVCGGKAIEGKGAFMKAGILTDIKRENPAYFE
B12526          TDLGPLSSVGARDQVVKQVEKAVQNGAKLVCGGKAIEGKGAFMKAGILTDIKRENPAYFE
B10988          TDLGPLSSVGARDQVVKQVEKAVQNGAKLVCGGKAIEGKGAFMKAGILTDIKRENPAYFE
CU1             TDLGPLSSVGARDQVVKQVEKAVQNGAKLVCGGKAIEGKGAFMKAGILTDIKRENPAYFE
CU1rif2         TDLGPLSSVGARDQVVKQVEKAVQNGAKLVCGGKAIEGKGAFMKAGILTDIKRENPAYFE
uvs51           TDLGPLSSVGARDQVVKQVEKAVQNGAKLVCGGKAIEGKGAFMKAGILTDIKRENPAYFE
PROIMI          TDLGPLSSVGARDQVVKQVEKAVQNGAKLVCGGKAIEGKGAFMKAGILTDIKRENPAYFE
NCIMB11163      TDLGPLSSVGARDQVVKQVEKAVQNGAKLVCGGKAIEGKGAFMKAGILTDIKRENPAYFE
                * *.**** .****:* ******.***.*******::  ****:*****:*.*:******
```

```
pomaceae        EFFGPIAQIYSVESEAKAIELANDSPYGLGGAVFAKDIEHGCKVAEQIETGMVSVNKPLW
francensis      EFFGPVAHVYAVKDEAAAIELANDSPYGLGGAVFAPDLDKGREVAEQIETGMVAINKPLW
Z6              EFFGPIAQIYAVKDEAEAIELANDSPYGLGGAVFAPDVEQGRKVAEQIETGMVAINKPLW
B23394          EFFGPIAQIYAVKDEAEAIELANDSPYGLGGAVFAPDVEQGRKVAEQIETGMVAINKPLW
B4492           EFFGPIAQIYAVKDEAEAIELANDSPYGLGGAVFAPDVEQGRKVAEQIETGMVAINKPLW
ZM4             EFFGPIAQIYAVKDEAEAIELANDSPYGLGGAVFAPDVEQGRKVAEQIETGMVAINKPLW
CP1             EFFGPIAQIYAVKDEAEAIELANDSPYGLGGAVFAPDVEQGRKVAEQIETGMVAINKPLW
B1960           EFFGPIAQIYAVKDEAEAIELANDSPYGLGGAVFAPDVEQGRKVAEQIETGMVAINKPLW
CP3             EFFGPIAQIYAVKDEAEAIELANDSPYGLGGAVFAPDVEQGRKVAEQIETGMVAINKPLW
CP4             EFFGPIAQIYAVKDEAEAIELANDSPYGLGGAVFAPDVEQGRKVAEQIETGMVAINKPLW
B12526          EFFGPIAQIYAVKDEAEAIELANDSPYGLGGAVFAPDVEQGRKVAEQIETGMVAINKPLW
B10988          EFFGPIAQIYAVKDEAEAIELANDSPYGLGGAVFAPDVEQGRKVAEQIETGMVAINKPLW
CU1             EFFGPIAQIYAVKDEAEAIELANDSPYGLGGAVFAPDVEQGRKVAEQIETGMVAINKPLW
CU1rif2         EFFGPIAQIYAVKDEAEAIELANDSPYGLGGAVFAPDVEQGRKVAEQIETGMVAINKPLW
uvs51           EFFGPIAQIYAVKDEAEAIELANDSPYGLGGAVFAPDVEQGRKVAEQIETGMVAINKPLW
PROIMI          EFFGPIAQIYAVKDEAEAIELANDSPYGLGGAVFAPDVEQGRKVAEQIETGMVAINKPLW
NCIMB11163      EFFGPIAQIYAVKDEAEAIELANDSPYGLGGAVFAPDVEQGRKVAEQIETGMVAINKPLW
                *****:*::*:*:.** ****************** *:::* :**********::*****

pomaceae        TAPELPFGGVKHSGYGRELSHFGIQEFINWKLIDASAAE
francensis      TAPELPFGGVKHSGYGRELSHFGIQEFINWKLIDGSAA-
Z6              TAPELPFGGVKHSGYGRELSHFGIQEFINWKLIDASAA-
B23394          TAPELPFGGVKHSGYGRELSHFGIQEFINWKLIDASAA-
B4492           TAPELPFGGVKHSGYGRELSHFGIQEFINWKLIDASAA-
ZM4             TAPELPFGGVKHSGYGRELSHFGIQEFINWKLIDASAA-
CP1             TAPELPFGGVKHSGYGRELSHFGIQEFINWKLIDASAA-
B1960           TAPELPFGGVKHSGYGRELSHFGIQEFINWKLIDASAA-
CP3             TAPELPFGGVKHSGYGRELSHFGIQEFINWKLIDASAA-
CP4             TAPELPFGGVKHSGYGRELSHFGIQEFINWKLIDASAA-
B12526          TAPELPFGGVKHSGYGRELSHFGIQEFINWKLIDASAA-
B10988          TAPELPFGGVKHSGYGRELSHFGIQEFINWKLIDASAA-
CU1             TAPELPFGGVKHSGYGRELSHFGIQEFINWKLIDASAA-
CU1rif2         TAPELPFGGVKHSGYGRELSHFGIQEFINWKLIDASAA-
uvs51           TAPELPFGGVKHSGYGRELSHFGIQEFINWKLIDASAA-
PROIMI          TAPELPFGGVKHSGYGRELSHFGIQEFINWKLIDASAA-
NCIMB11163      TAPELPFGGVKHSGYGRELSHFGIQEFINWKLIDASAA-
                **********************************.***
```

Figure S7. *Z. mobilis* AldB (ZMO1754) multiple sequence alignment. Alignment of AldB from different laboratory strains of *Zymomonas mobilis* was performed by MUSCLE. Origin and characteristics of different laboratory strains of *Z. mobilis* are described in (7).
